# Use of a pacemaker magnet to remove a broken suture needle during a laparoscopic cholecystectomy

**DOI:** 10.1093/jscr/rjab444

**Published:** 2021-10-12

**Authors:** Derek Titus, James Rifenbery

**Affiliations:** Western University of Health Sciences, College of Osteopathic Medicine of the Pacific-Northwest, Lebanon, OR, USA; Department of General Surgery, Virginia Mason Franciscan Health, Seattle, WA, USA; Western University of Health Sciences, College of Osteopathic Medicine of the Pacific-Northwest, Lebanon, OR, USA; Department of General Surgery, Virginia Mason Franciscan Health, Seattle, WA, USA; Soundview Ambulatory Surgery Center, Gig Harbor, WA, USA

## Abstract

Prompt removal of unintended surgical foreign bodies is essential for prevention of adverse consequences of retained surgical foreign bodies postop. Current practices utilizing radiographic images in combination with visual inspection and palpation to remove foreign bodies can lead to increased surgical times and tissue damage. A suture needle fragment broke off during a laparoscopic cholecystectomy performed on a morbidly obese adult female. After being unable to retrieve the fragment by increasing the midline incision by ~7 cm and undergoing an additional ~1 of intraoperative time, a pacemaker magnet wrapped in sterilized plastic was able to instantaneously remove the shard. Techniques utilizing magnetism could be researched and developed for the removal of surgical foreign bodies both intraoperatively and postop.

## INTRODUCTION

Retained surgical foreign bodies (RSFB) are classified as one of 29 ‘never events’ requiring mandatory reporting [1]. It is estimated that over half of RSFB cases occur during abdominal procedures with an incidence of 0.3–1.0 cases per 1000 abdominal operations. Risk factors include an increased procedure complexity, emergent operations, increased patient body mass index and prolonged surgeries [2]. They have the potential to cause significant harm to patients while generating substantial social and legal issues for medical parties held responsible. Even though many patients are asymptomatic after RSFB events, some have been found to report pain, nausea, vomiting, abscesses, wound complications and masses requiring further workup. Responsible medical parties face issues involving a damaged reputation along with estimated medicolegal expenses ranging from $37 000 to $2 350 000 per incidence [3].

Due to the serious ramifications of RSFB, guidelines produced by the Association of Perioperative Registered Nurses dictate that unintended foreign objects recognized must be removed before a surgery can conclude. The current approach for foreign object removal consists of systemic visual inspections in combination with intraoperative X-rays for localization and subsequent removal. Since abdominal X-rays have been found to have a low specificity and sensitivity for small needles, this approach can lead to increased operative times with the potential for organ injury [4]. Here, we present a case where a pacemaker magnet was implemented intraoperatively to remove a broken suture needle fragment.

## CASE REPORT

The patient presented as a morbidly obese adult female with a several hour history of constant postprandial severe right upper quadrant pain (RUQ). Physical examination demonstrated RUQ tenderness with guarding and a positive Murphy’s sign. Lab results confirmed elevated direct bilirubin levels and RUQ ultrasound findings demonstrated a markedly distended gallbladder with gallstones present. These findings generated a primary differential diagnosis of acute cholecystitis with a treatment plan of performing a laparoscopic cholecystectomy.

During the procedure, a size 0 taper point UR-6 Vicryl synthetic absorbable suture was utilized to close the fascia incision made at midline just superior the umbilicus. This maneuver caused the needle to break apart into two distinct fragments with the free shard getting lodged into the surrounding adipose tissue. In order to try to retrieve this shard, the surgeon increased the midline skin incision from ~3 to ~10 cm and set up two hemostats to serve as markers to localize the shard. Subsequent abdominal radiography images of the incisional region were taken utilizing a C-arm that had been used prior to take an intraoperative cholangiogram. The surgeon then observed where the needle fragment was located in relation to the hemostats and attempted to find it through palpation. Hemostats were moved and additional images were taken as needed for better localization.

After this process of moving hemostats, taking an image with the C-arm and palpating to find the needle fragment had extended the surgery by an additional ~1 h, the case anesthesiologist relayed a potential solution to the dilemma. A pacemaker magnet was then inserted into a sterilized plastic bag and slowly lowered into the open wound. The lost fragment instantaneously attached itself to the covered magnet and was liberated from the wound without any further complications.

## DISCUSSION

Approaches using radiographic images in combination with visual inspection and palpation to remove unintended foreign objects during surgery have been shown to be nonideal. In the current described case, this method resulted in an increased incision size of ~7 cm along with an extra ~1 h of intraoperative time. The broken needle fragment was only able to be removed leading to the end of the procedure after a nonconventional approach of implementing a simple pacemaker magnet was utilized. Others have found similar success in using magnets to remove foreign objects with one group even developing a laparoscopic magnetic device [4] and another enacting a similar approach to remove sharp iron objects swallowed by a psychiatric patient [5].

Taken together, this suggests the need for continued research into developing magnetic approaches for the removal of unintended metal foreign bodies both intraoperatively and in the setting of follow-up procedures to remove accidental RSFB found postop. This could help reduce extended operation times and tissue damages due to these events.

## FUNDING

The authors report no external sources of funding for the current study.

## CONFLICT OF INTEREST STATEMENT

The authors report no conflicts of interest for the current study.
